# Improving the diagnosis of acute ischemic stroke on non-contrast CT using deep learning: a multicenter study

**DOI:** 10.1186/s13244-022-01331-3

**Published:** 2022-12-06

**Authors:** Weidao Chen, Jiangfen Wu, Ren Wei, Shuang Wu, Chen Xia, Dawei Wang, Daliang Liu, Longmei Zheng, Tianyu Zou, Ruijiang Li, Xianrong Qi, Xiaotong Zhang

**Affiliations:** 1 Interdisciplinary Institute of Neuroscience and Technology, College of Biomedical Engineering and Instrument Science, Zhejiang University, Hangzhou, 310027 Zhejiang China; 2 Academy for Advanced Interdisciplinary Studies, Peking University, Beijing, China; 3 Infervision Institute of Research, Beijing, 100025 China; 4 Liaocheng People’s Hospital, Liaocheng, 252000 Shandong China; 5 Medical Imaging Center, Ankang Central Hospital, Ankang, 725000 Shanxi China; 6 Weihai Municipal Hospital, Weihai, 264200 Shandong China; 7 Department of Radiation Oncology, Stanford University School of Medicine, Stanford, CA 94304 USA; 8 School of Pharmaceutical Sciences, Peking University, Beijing, 100191 China; 9 Beijing Key Laboratory of Molecular Pharmaceutics and New Drug Delivery System, School of Pharmaceutical Sciences, Peking University, Beijing, 100191 China; 10 College of Electrical Engineering, Zhejiang University, Hangzhou, 310000 Zhejiang China; 11 MOE Frontier Science Center for Brain Science & Brain-machine Integration, Zhejiang University, Hangzhou, 310000 Zhejiang China

**Keywords:** Deep learning, Non-contrast computed tomography, Early ischemic changes, Alberta Stroke Program Early Computed Tomography Score, Multi-reader and multi-center study, Acute ischemic stroke

## Abstract

**Objective:**

This study aimed to develop a deep learning (DL) model to improve the diagnostic performance of EIC and ASPECTS in acute ischemic stroke (AIS).

**Methods:**

Acute ischemic stroke patients were retrospectively enrolled from 5 hospitals. We proposed a deep learning model to simultaneously segment the infarct and estimate ASPECTS automatically using baseline CT. The model performance of segmentation and ASPECTS scoring was evaluated using dice similarity coefficient (DSC) and ROC, respectively. Four raters participated in the multi-reader and multicenter (MRMC) experiment to fulfill the region-based ASPECTS reading under the assistance of the model or not. At last, sensitivity, specificity, interpretation time and interrater agreement were used to evaluate the raters’ reading performance.

**Results:**

In total, 1391 patients were enrolled for model development and 85 patients for external validation with onset to CT scanning time of 176.4 ± 93.6 min and NIHSS of 5 (IQR 2–10). The model achieved a DSC of 0.600 and 0.762 and an AUC of 0.876 (CI 0.846–0.907) and 0.729 (CI 0.679–0.779), in the internal and external validation set, respectively. The assistance of the DL model improved the raters’ average sensitivities and specificities from 0.254 (CI 0.22–0.26) and 0.896 (CI 0.884–0.907), to 0.333 (CI 0.301–0.345) and 0.915 (CI 0.904–0.926), respectively. The average interpretation time of the raters was reduced from 219.0 to 175.7 s (*p* = 0.035). Meanwhile, the interrater agreement increased from 0.741 to 0.980.

**Conclusions:**

With the assistance of our proposed DL model, radiologists got better performance in the detection of AIS lesions on NCCT.

**Supplementary Information:**

The online version contains supplementary material available at 10.1186/s13244-022-01331-3.

## Key points

- The model simultaneously segments infarcts and estimates ASPECTS by using baseline CT.
- A mirror-assembly module plus dual-path DCNN model improved the segmentation efficiency.
- We evaluated the model in a multi-reader and multicenter (MRMC) setting.

**Supplementary Information:**

The online version contains supplementary material available at 10.1186/s13244-022-01331-3.

## Introduction

Stroke is one of the major threats to human health, and it is the third leading cause of death in the world with high mortality and disability rate [1–3]. Non-contrast computed tomography (NCCT) is deemed as the first choice for all stroke diagnosis due to its relative high speed, broad accessibility and cost-effectiveness compared with magnetic resonance imaging (MRI) and CT perfusion (CTP) [3, 4], especially in the setting of emergence department [5]. NCCT not only has high sensitivity to the detection of intracranial hemorrhage, but also is a widely used tool to select patients for endovascular therapy [6–8]. The Alberta Stroke Program Early Computed Tomography Score (ASPECTS) is a quantitative score method for early ischemic changes (EIC) evaluation base on NCCT [9] and has been used in several randomized controlled trials (RCTs) for patient selection and exclusion [10–12]. Until now, NCCT-ASPECTS is still the most widely used modality for early ischemic triage and thrombolytic outcome prediction in emergency department [13–19].

However, EIC detection using NCCT is yet challenging and the ASPECTS can only be roughly determined in practice. The early signs of ischemia and their translation into ASPECTS suffer from considerable missed diagnosis and interrater variability due to the rater’s experience difference [20–22], since the mild infarction on NCCT is difficult to be recognized by naked eyes, and there is no obvious boundary among brain regions involved in ASPECTS scoring. As reported in the previous studies, only 10% acute ischemic stroke (AIS) and 7% hyperacute ischemic stroke patients could be detected by using NCCT only [5]. Moreover, the EIC detection is also experience-dependent and suffers from the limited inter-observer consistency [23, 24]. More endeavor needs to be devoted to improve the EIC detection sensitivity and ASPECTS assessment consistency.

Several software applications using artificial intelligence, including classical machine learning and deep learning, have been designed for automated EIC detection and ASPECTS scoring [25–29]. The classical machine learning approach usually uses the image grayscale-based segmentation and pre-defined features to define the lesion, e.g., e-ASPECTS [29] (Brainomix, UK), RAPID-ASPECTS [24] (iSchemaView, USA) and Frontier ASPECTS [30] (Siemens Healthcare, Germany). These methods are limited by hand-crafted texture patterns or geometric shapes that rely on data scientists’ expertise, and thus the ASPECTS results vary among studies [30, 31]. Deep learning (DL) has emerged to be a powerful technique in medical imaging diagnosis, which can discover abstract task-specific features and further uses these features to produce accurate clinical interpretations in an end-to-end manner [32–35]. The application of DL methods in the EIC detection and ASPECTS interpretation has just emerged [27, 28] and showed good performance. However, there are few well-established DL approaches for NCCT-ASPECTS scoring and most of the existing studies are single-centered; in addition, they only focus on the evaluation of the model efficiency rather than the model performance in clinical emergency scenarios. Therefore, the DL model performance in clinical scenarios needs further investigations.

To address the above issues, we propose a novel DL model for the automatic AIS lesions detection and ASPECTS scoring and further demonstrate the value of the model in clinical assistance. Firstly, the model is developed based upon a reasonably large NCCT dataset across 5 stroke centers. Secondly, the model uses a mirror assembly module and a dual-path DCNN model to enhance the lesion detection ability. Besides, to validate the model clinical performance, we utilize a multi-reader and multicenter (MRMC) experiment to evaluate the radiologists’ diagnosis under the aid of the proposed model.

## Methods and materials

This retrospective study was approved by the Ethics Review Board of all participating hospitals. Patients’ private information in the Digital Imaging and Communications in Medicine (DICOM) header file was desensitized. Owing to the retrospective nature, the requirement for informed consent was waived in this study.

### Participants

**Fig. 1:**
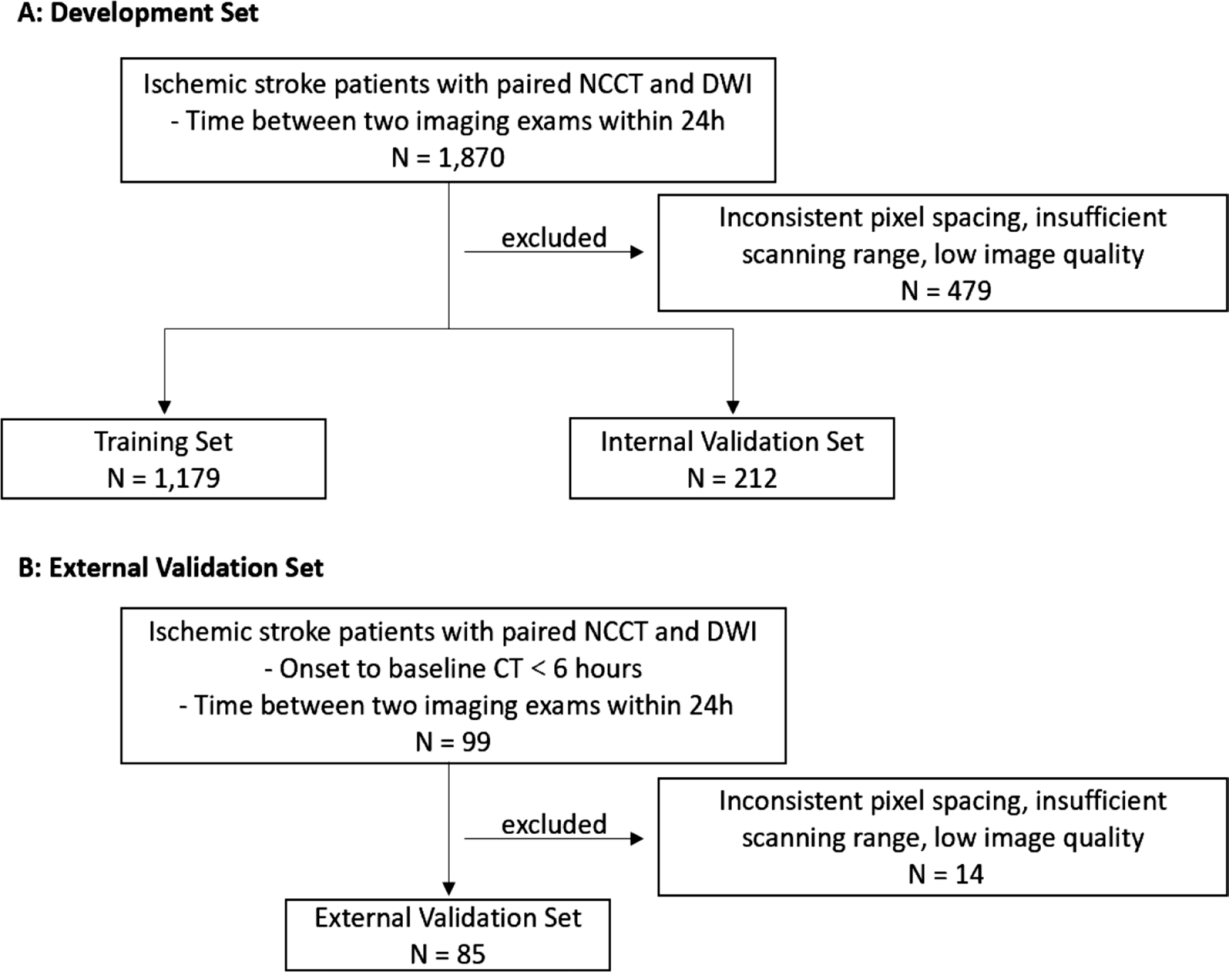
Flowchart of data inclusion

The Development Set was acquired by searching for the keyword of “acute ischemic stroke” in the radiology information system (RIS) between 2013 and 2017 from two participating hospitals, and AIS patients with NCCT-to-DWI time < 24 h were included in this dataset (1870 patients); then, images with inconsistent pixel spacing, insufficient scanning range or low image quality were excluded. The development set was divided into a training set and an internal validation set at a ratio of 5:1, as illustrated in Fig. 1.

The external validation set was curated from the other three hospitals. AIS patients with onset-to-NCCT time < 6 h and NCCT-to-DWI time < 24 h were included (99 patients). The unqualified images were excluded, and the clinical characteristics of the enrolled patients were also collected.

Specifically, only MCA territory infarcts were included to be analyzed in both the development set and the external validation set. The image acquisition parameters are provided in Additional file 1.

### Ground truth determination

Ground truth of ischemic region and ASPECTS for both the development set and the external validation set were established for each NCCT scan via the consensus of three board-certified radiologists (Z.T.Z., L.C.F., P.F.), and all the radiologists had at least 10-year experience in emergency neuroradiology (these experts received guiding instructions on www.aspectsinstroke.com prior to labeling). The ischemic regions were manually drawn and scored by one radiologist and validated by the other two radiologists independently. All the NCCT ischemic regions were delineated using 3D Slicer (version 4.8.1, www.slicer.org) with reference to the paired DWI images and radiology reports. ASPECTS scores for each region were also rated according to ASPECTS guidelines.

### DL model development

**Fig. 2:**
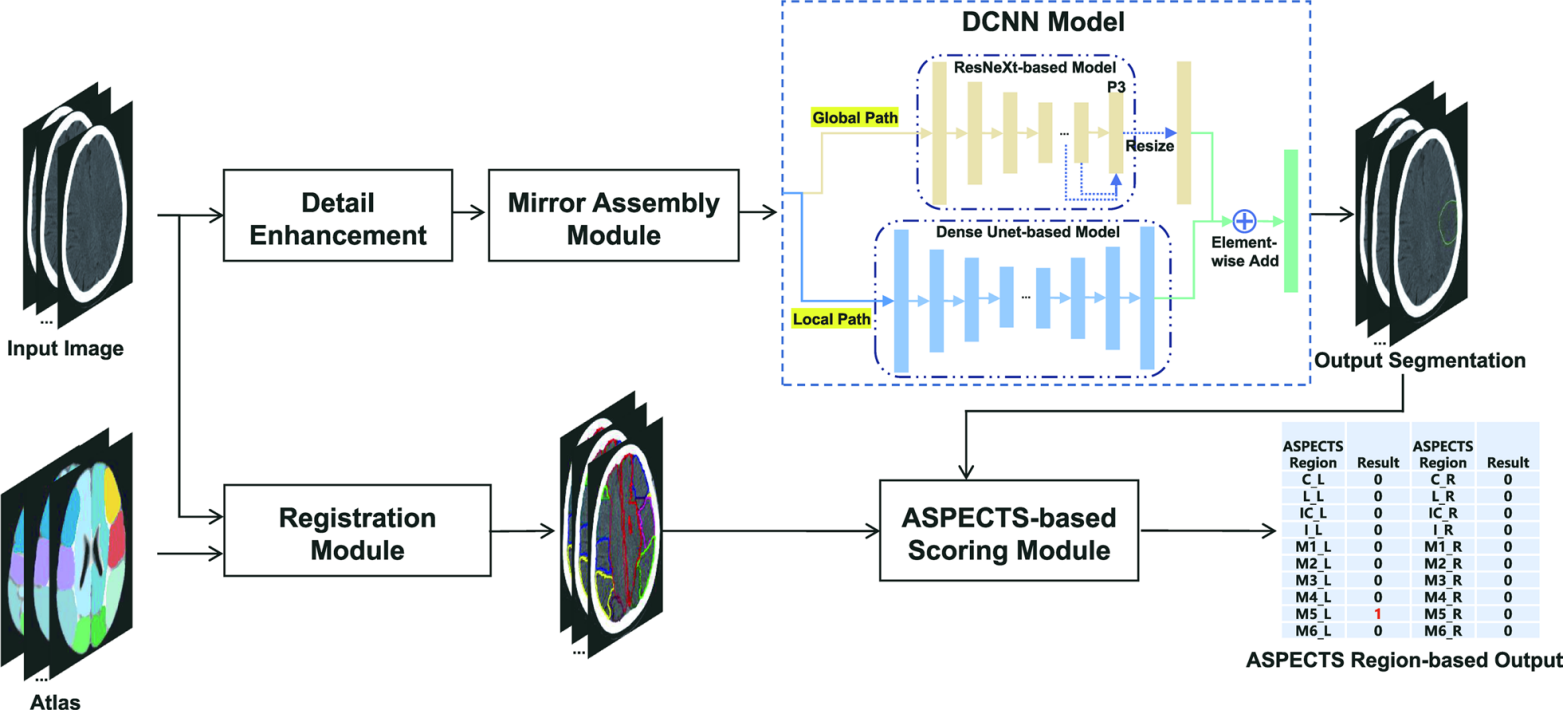
Overview of the proposed DL model for lesion segmentation and ASPECTS scoring. This model included five key procedures of detail enhancement, mirror assembly module, DCNN model, linear registration and ASPECTS scoring module. The model generated two types of data: ischemic lesion segmentation and region-based ASPECTS

The proposed DL model is shown in Fig. 2, consisting of 5 key procedures to score the ASPECTS automatically. To reduce the influence of noise, we firstly pre-processed the NCCT images with median filter and the dark image enhancement algorithm [36] (“Detail Enhancement”). The manifestations of EIC on NCCT of AIS patients are extremely insignificant, and experts can only make decisions based on the slight gray-level difference between the left and right brains in NCCT images. For this reason, we designed a “Mirror Assembly Module,” which obtained the mirror image by flipping the original NCCT image around the midline of the brain, and used the original NCCT image to subtract the mirrored one to obtain the difference image. Then, we spliced the original images, the mirror images and the difference images to obtain the input samples of our DL model.

Specifically, the boundaries of acute ischemic lesions were not obvious in the NCCT images and hard to be delineated accurately, thus they were easy to be misdiagnosed. Therefore, if such noisy annotations were used for model training, it would lead to model learning bias and damage model efficiency, especially when the traditional panoptic segmentation methods, like UNet [37] and panoptic FPN [38], were directly applied for model development. In order to tackle this problem and being inspired by the idea of ensemble learning, we proposed a novel deep convolutional neural network (DCNN) model with two pathways—“Global Path” and “Local Path.” “Global Path” was built based on ResNeXt-model [39, 40] to achieve good global localization and coarse segmentation of ischemic lesions and reduce the lesion-level missed diagnosis rate. However, “Global Path” could only output the coarse segmentation images from the P3 layer of ResNeXt-50 and ignored the boundary details of ischemic lesions, thus we developed a "Local Path" as a supplement to “Global Path” to perform careful pixel-level segmentation under full-resolution images based on the Dense-UNet model [41]. Then, we resized the output segmentations of “Global path” and “Local Path” to the size of origin NCCT images with bilinear interpolation and subsequently fused the resized two-pathway output segmentation by element-wise addition to obtain the final output segmentation. Details of the “Global Path” and “Local Path” are provided in Additional file 1.

In order to score the ASPECTS, we generated blood supply maps based on the MNI brain template [42, 43], as shown in Additional file 1: Fig. S3. Then, by linearly registering the blood supply region maps to the NCCT images (“Linearly Registration”), the blood supply regions of the corresponding individuals were obtained. At last, according to the ASPECTS scoring criteria [9] of calculating the overlap proportion of the lesion segmentation and the blood supply regions, a score for each ASPECTS region was obtained (“ASPECTS Scoring Module”). The proposed model was implemented by using Python 2.7 and the MxNet framework (version 1.4.0, mxnet.apache.org) with two Nvidia GTX 1080Ti GPUs for computation accelerations. The model was trained with the stochastic gradient descent optimizer and a stepped power-decaying learning rate scheduler starting from 0.002. The deep learning model was trained with an input image size of 512 × 512 and 100 epochs. When the model loss of the internal validation set began to increase, the training process was stopped. In order to make the model focus on the details as well as the overall segmentation contour of the lesion, we constructed a loss function integrating the cross-entropy and DICE loss functions. The loss function is introduced in Additional file 1.

### Multi-reader and multicenter (MRMC) experiment

We conducted a MRMC experiment on the external validation set to evaluate the clinical efficacy of the proposed DL model in the help of four radiologist raters from different tertiary teaching hospitals; each rater had over 10 years of clinical experience in emergency radiology. Before the experiment, all raters were asked to participate in an educational session on how to use ASPECTS according to the instructions on www.aspectsinstroke.com, and then each of them was provided with apparatus like the ones equipped at the emergency department. They were also instructed to interpret each scan in line with the emergency clinical practice. The raters were blind with the patients’ medical histories and onset symptoms. Two sessions were operated in this MRMC experiment: in the first session, only original scans were available for rater review. And the second session was performed after a washout period of 2 weeks and the model segmentation and region-based ASPECTS were provided to the raters. All raters were asked to score the region-based ASPECTS on a spreadsheet, and their reading time was also recorded in the two sessions. Interrater agreement among the four raters was also calculated and compared between the two sessions.

### Statistical analysis

Baseline demographic characteristics are demonstrated in Table 1. DL models were evaluated in a region-based manner according to the ASPECTS criteria. Twenty binomial outcomes (20 ASPECTS regions) were generated for each case. The ROC curve was plotted in the following manner: we enumerated the probability thresholds for the DCNN model between 0 and 1, and for each threshold, the corresponding TPR and FPR were calculated based on the model outputs for each ASPECTS region; these TPR-FPR pairs were plotted and formed into ROC curves. AUC (area under curve) was calculated based on the ROC curve, and its confidence interval (CI) was calculated referenced to Dai Feng’s method [44]. Dice similarity coefficient (DSC), precision and recall were used for segmentation performance evaluation.

**Table 1:** Baseline demographic and clinical characteristics

	Training set	Internal validation set	External validation set	*p* value
	(*n* = 1179)	(*n* = 212)	(*n* = 85)	
*Patient characteristics*				
Age, mean ± SD, years	61.3 ± 12.5	62.0 ± 11.1	66.6 ± 12.4	< .001
Male	70.80%	72.20%	64.70%	0.12
	(835/1179)	(153/221)	(55/85)	
*Manufacturer*				< .001
GE	57.00%	61.80%	28.20%	
	(672/1179)	(131/212)	(24/85)	
PHILIPS	1.10%	0.90%	58.80%	
	(13/1179)	(2/212)	(50/85)	
SIEMENS	41.70%	37.3% (79/212)	12.90%	
	(492/1179)		(11/85)	
Other	0.20%	0.00%	0.00%	
	(2/1179)	(0/212)	(0/85)	
Time from onset to CT (min)	–	–	176.4 ± 93.6	–
Admission NIHSS score	–	–	5(2–10)	–
			(*n* = 76/85)	
Admission systolic BP (mmHg)	–	–	153 ± 22	–
			(*n* = 84/85)	
*Risk factors*				
Previous stroke	–	–	14.10%	–
			(12/85)	
Hypertension	–	–	54.10%	–
			(46/85)	
Diabetes	–	–	16.50%	–
			(14/85)	
Smoking	–	–	24.70%	–
			(21/85)	
Atrial fibrillation	–	–	15.30%	–
			(13/85)	
CVD	–	–	17.60%	–
			(15/85)	

Data are percentages with numbers in parentheses for categorical variables. Continuous variables are reported as mean ± standard deviation with number of entries in parentheses. Ordinal variables (NIHSS score) are reported as median with interquartile range (IQR) and number of entries in parentheses. ANOVA was used as the test for the difference of mean for continuous variables. Fisher’s exact test was used to test for the difference of proportion for categorical variables. The post hoc analysis was performed by Tukey’s HSD method. Other manufacturers were omitted from hypothetical tests due to the absence of data

NIHSS, National Institutes of Health Stroke Scale; BP, Blood pressure; CVD, cardiovascular disease

In MRMC experiment, we used the region-based sensitivity and specificity for clinical efficacy evaluation. The total time was recorded for each session, and the averaged case-reading time was calculated through averaging the total duration by the total number of cases. The averaged time was compared with paired Student's t test. The sensitivity and specificity were compared with the McNemar test. The interrater agreement was measured using ICC (intraclass correlation coefficient) of two-way mixed single absolute agreement [45]. All statistical analysis was performed by R programming language (version 3.6.2), and a two-sided *α* < 0.05 was considered statistically significant.

## Results

### Patient data

At last, a total of 1,391 patients (1,179 patients in the training set and 212 patients in the internal validation set) were enrolled for model development and 85 patients were eligible for model external validation (see Fig. 1). All eligible patients’ baseline characteristics are summarized in Table 1. The age difference among these three sets was statistically significant (*p* < 0.001), and there was no gender difference (*p* = 0.120); furthermore, post hoc analysis of age showed that there was no difference between the training set and the internal validation set, and a statistical age difference was found between the two groups and the external validation set. It was observed that CT scanners made by GE Healthcare (Boston, MA, USA) and Siemens Healthineers (Erlangen, Germany) were in a majority for development set (Training Set: GE 57.0%, SIEMENS 41.7%; Internal Validation Set: GE 61.8%, SIEMENS: 37.3%), while PHILIPS Healthcare (Best, The Netherlands) was the major vendor in the external validation set (58.8%), and there was a significant manufacturer difference among the three groups (*p* < 0.001).

**Fig. 3:**
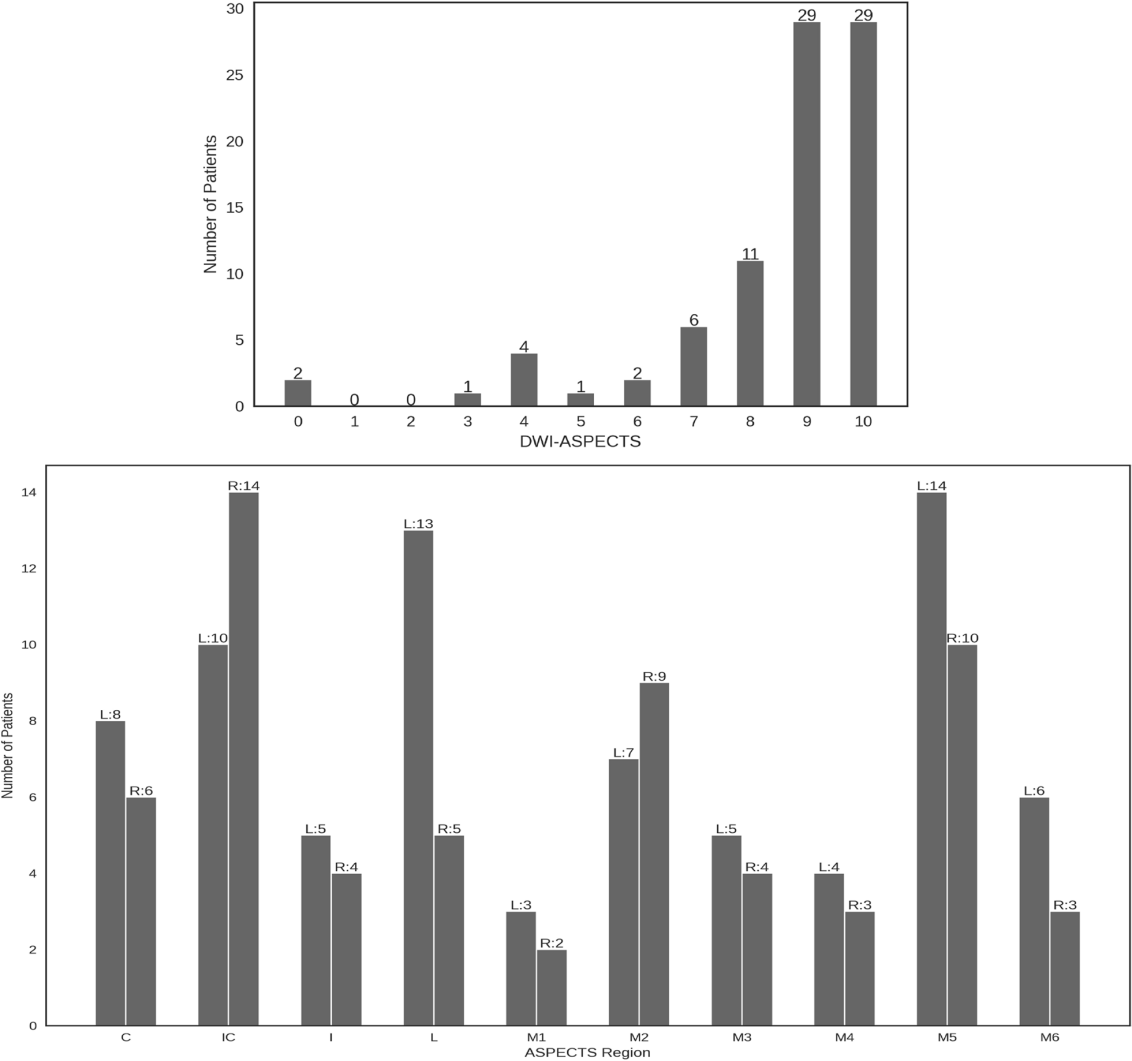
DWI-ASPECTS and the ASPECTS-Region distribution of the external validation set. C, Caudate; IC, Internal capsule; I, Insula; L, Lentiform; M1, frontal operculum; M2, anterior temporal lobe; M3, posterior temporal lobe; M4, anterior MCA; M5, lateral MCA; M6, posterior MCA

Detailed clinical information was only recorded for the external validation set. The mean time from onset to baseline CT was 176.4 ± 93.6 min, and the median NIH Stroke Scale (NIHSS) was 5 (IQR 2–10). The DWI-ASPECTS distribution and the ASPECTS region of the external validation set are illustrated in Fig. 3 with a bar graph (median DWI-ASPECTS = 9, IQR 8–10). In total, 1700 (85 × 20) ASEPECTS regions in the external validation set were scored.

### The DL model efficiency

**Fig. 4:**
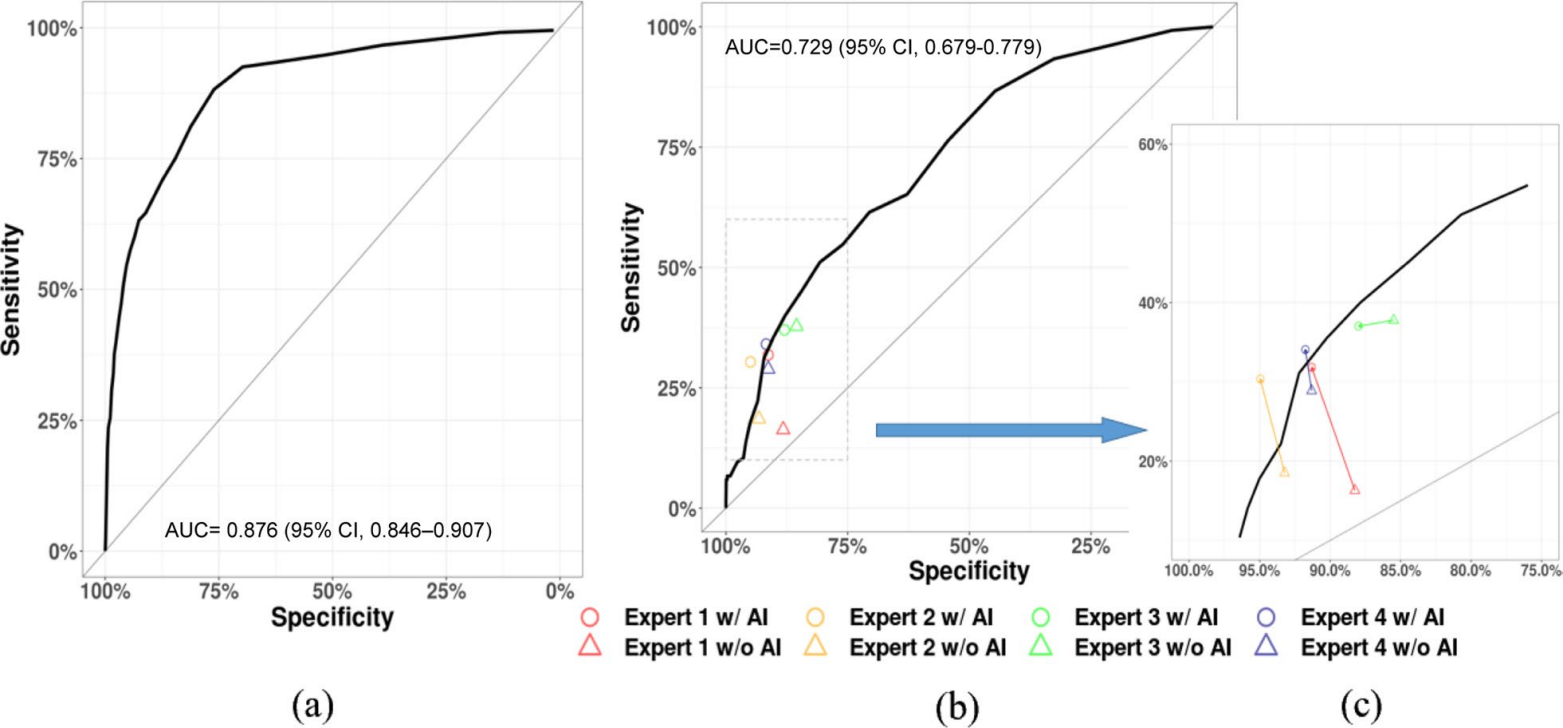
Performance of the proposed DL-based model. **a** ROC curve on region-based ASPECTS analysis (20 regions) for the DL-based model on the internal validation set. **b** ROC curve on region-based ASPECTS analysis for the DL-based model on the external validation set, and the performance of the four raters was also depicted using circle or triangle. **c** Enlarged illustration of the rater performance in (**b**)

ROC curves of the internal and external validation sets are shown in Fig. 4a, b). For the internal validation set, the model achieved an AUC of 0.876 (95% CI 0.846–0.907), while on the external validation set, the model achieved an AUC of 0.729 (95% CI 0.679–0.779). The external validation showed that the trained model provided higher sensitivity and specificity in identifying individual ischemia ASPECTS regions than all expert raters. The DSC, precision and recall was 0.600, 0.528 and 0.694 for the internal validation set and 0.762, 1.000 and 0.616 for the external validation set, respectively.

**Fig. 5:**
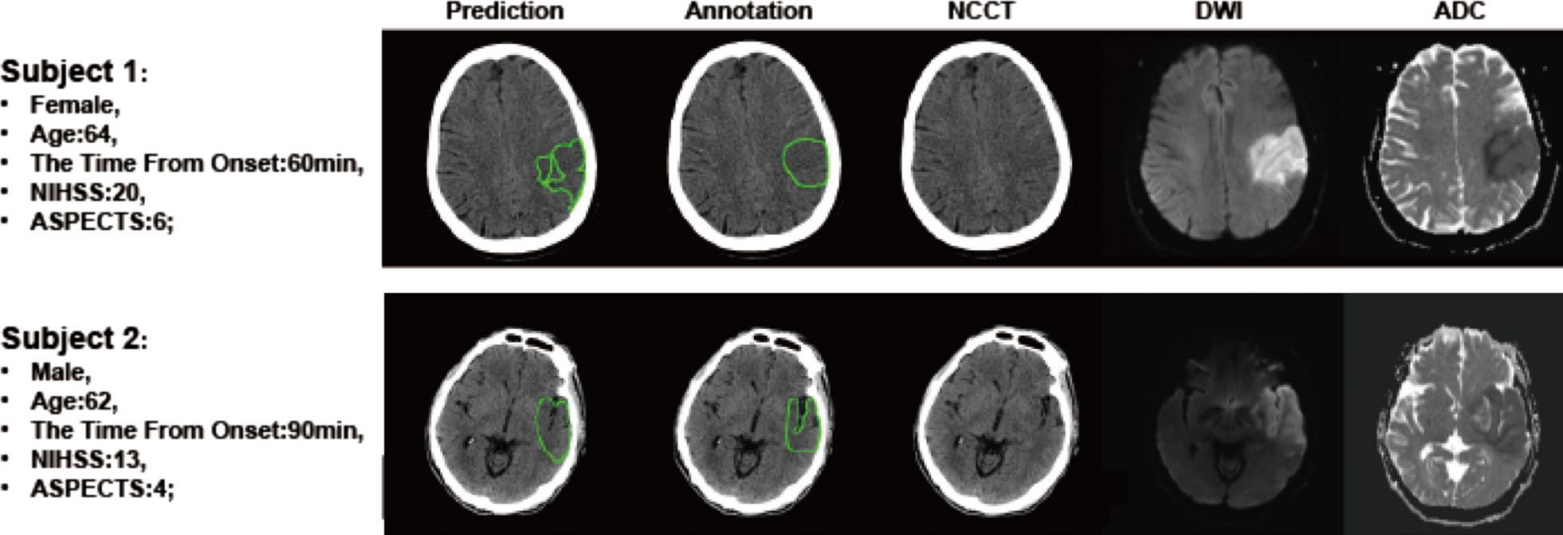
Visualization cases of the DL-based model. The prediction is the model segmentation output and the annotation denotes the ground truth defined by the expert radiologists. The dice, precision and recall were 0.817, 1.0 and 0.690 for subject 1 and 0.670, 1.0 and 0.502 for subject 2, respectively

To visualize the model performance, NCCT segmentation result of two patients is presented in Fig. 5. Obviously, it is confounded to define the lesion boundary with naked eyes, whereas the model could almost achieve this goal.

### Performance comparisons of the MRMC study

The performance of the expert raters on the external validation set is summarized in Table 2. The average sensitivity was 0.254 (95% CI 0.22–0.26) and 0.333 (95% CI 0.301–0.345), and the specificity was 0.896 (95% CI 0.884–0.907) and 0.915 (95% CI 0.904–0.926) without vs. with AI, respectively—there was a statistical difference for average specificity (*p* = 0.014) but not for the average sensitivity (*p* = 0.196). As shown in Fig. 4b, c, with the assistance of the DL model, the sensitivity and specificity of Expert 1 and 2 were significantly improved (Expert 1: sensitivity *p* < 0.001, specificity *p* = 0.002; Expert 2: sensitivity *p* = 0.006, specificity *p* = 0.022); for Expert 3, the specificity was significant improved (*p* = 0.019) and the sensitivity behaved no significant difference (*p* = 1); no significant differences were observed in the sensitivity (*p* = 0.281) and specificity (*p* = 0.630) of Expert 4. In addition, the region-based ICC with model aid among the four raters comes up to 0.980 compared to 0.741 without model aid, and the score-based ASPCETS ICC improved from 0.614 to 0.809.

**Table 2:** Performance of the raters on external validation set

	Baseline	Aided	*p* value
*Expert 1*			
Sensitivity	0.163 (0.105, 0.236)	0.319 (0.241, 0.404)	< .001
Specificity	0.882 (0.865, 0.898)	0.913 (0.898, 0.927)	0.002
*Expert 2*			
Sensitivity	0.185 (0.124, 0.261)	0.304 (0.228, 0.389)	0.006
Specificity	0.932 (0.919, 0.944)	0.950 (0.937, 0.960)	0.022
*Expert 3*			
Sensitivity	0.378 (0.296, 0.465)	0.370 (0.289, 0.458)	1
Specificity	0.855 (0.837, 0.872)	0.880 (0.863, 0.896)	0.019
*Expert 4*			
Sensitivity	0.289 (0.214, 0.373)	0.341 (0.261, 0.427)	0.281
Specificity	0.913 (0.898, 0.927)	0.918 (0.903, 0.931)	0.63
*Average*			
Sensitivity	0.254 (0.167, 0.341)	0.333 (0.248, 0.419)	0.196
Specificity	0.896 (0.884, 0.907)	0.915 (0.904, 0.926)	0.014
Region-based ICC	0.741	0.98	–
Score-based ICC	0.614	0.809	–

Sensitivity and specificity were expressed as point estimate with lower and upper bound of 95% confidence interval (CI) in parentheses. CI for average sensitivity and specificity was calculated by the Jackknife method

Regarding the reading time, the average reading time of the external validation set is summarized in Table 3. Obviously, with the aid of the DL model, the interpretation time of all expert raters was significantly reduced (*p* = 0.035).

**Table 3:** Average time per case in reading experiment

	Average time per case (*n* = 85)		
	Baseline (s)	Aided (s)	*p* value
Expert 1	202.1	176.2	–
Expert 2	250.1	176.8	–
Expert 3	197.7	176.2	–
Expert 4	226.1	173.7	–
Average	219.0 ± 24.2	175.7 ± 1.4	0.035

Total time for each reader in each session was recorded and the averaged case-reading time was calculated. Average time for each session was expressed in mean ± standard deviation and was compared with paired Student’s *t* test

## Discussion

Diagnosis of EIC and ASPECTS using NCCT is a persistent challenge in emergency department with urgent clinical needs. This study attempts to utilize the DL technique to detect infarction area and evaluate the DL performance in emergency clinical scenario. Specifically, our proposed DL model demonstrates good efficacy in ASPECTS scoring and achieves an AUC of 0.729 in the external validation set. The DL model using mirror assembly module and dual-path DCNN model obtains a high DSC of 0.762 in occult lesion segmentation. With the model assistance, raters show improved performance in ASPECTS scoring with higher sensitivity and specificity, shorter operating time and good interrater agreement. To the best of our knowledge, it is the first time to investigate the emergency efficiency of the DL model in a MRMC manner.

Our proposed DL model outperforms the previously reported studies [27, 46] in lesion segmentation, and several factors lay the foundation of the good performance: firstly, the high-quality ground truth provided by stroke experts and follow-up DWI, which assures the trained DL model to learn the task-specific feature effectively; secondly, the DL method is good at simulating and integrating the experience of stroke experts in detecting AIS lesions; lastly but most importantly, the innovative model pipeline that comprises a mirror assembly module to capture the image difference between left and right brains and a dual-path DCNN model to tackle the problems of indiscernible lesion detection and segmentation. This pipeline could enhance the feature characteristics associated with image segmentation tasks while suppressing redundant features. In addition, to score the ASPECTS accurately, we also develop an ASPECTS atlas and register it to the original NCCT images reversely to reduce image deformation errors; furthermore, in order to suppress the segmentation errors, we use the region-level ASPECTS to determine the segmentation threshold rather than DSC. EIC detection and ASPECTS scoring on NCCT is clinically desired, but the subtle signs of EIC cannot always be captured visually, as shown in Fig. 5. Therefore, the efficacy of deep learning in indiscernible features detection may be an accelerator to the NCCT clinical application in AIS diagnosis.

Performance comparisons between radiologist and automatic software in ASPECTS scoring have been reported previously [25, 26, 29], whereas our study differed from previous ones in using the AI model as first-reader. AI as first-reader has been widely accepted in pulmonary nodules detection [47], but fewer have been reported in NCCT-ASPECTS interpretation. As shown in Fig. 4b, c and Table 2, all the stroke experts aided with the DL model reached a relatively high sensitivity level (sensitivity ≥ 0.3) with improved specificities (*p* = 0.014), along with the reduced reading time (*p* = 0.035). However, we also note that not all raters’ sensitivities or specificities are statistically significant, which may attribute to the raters’ acceptance of the model result. Since the signs are too faint to be observed, the confidence for lesion determination may vary with the readers’ experience. Various studies have shown that only modest to moderate interrater agreement was achieved for determining NCCT-ASPECTS, and the ICC of radiologists ranged from 0.579–0.936 [21, 23, 24, 28, 48, 49]. In contrast, in our study, the ASPECTS reliability (ICC 0.980) of the radiologists with AI assistance is significantly increased and help to improve the diagnostic confidence and medical quality consistency. In a word, the AI model can be a valuable supplement and/or confirmation to the expert interpretation in ASPECTS scoring with improved performance and reliability.

The greatly varied expert diagnosis and software performance in NCCT-ASPECTS evaluation remained to be a concern, as reported sensitivity and specificity ranged from 0.26 to 0.8 and 0.87 to 0.97, respectively [5, 25, 26, 28, 29]. In our study, the resulting sensitivity and specificity are not superior to previous reported methods, which can be attributed to the experiment setting of the external validation set, including the severity of the infarction, the distribution of the ASPECTS and the invaded-region, the radiologists’ experience and the varied scan vendors. Firstly, compared to previous studies, enrolled patients in our external validation set have lower NIHSS (5, 2–10) and age (66.6 ± 12.3), making the lesion more blurred to be detected compared to that of Masaki Naganuma’ study [28]. Secondly, the lesion detection sensitivity is greatly affected by the ASPECTS distribution, especially for the super acute stroke with ASPECTS > 7, while the median ASPECTS of this study is only 9 (IQR 8–10) with 29 patients of ASPECTS 9 and 29 patients of ASPECTS 10, respectively, raising the difficulty of lesion detection compared to that of Hulin Kuang’ study [50]. In addition, the ASPECTS accuracy could also be affected by brain region distribution [51]. As shown in Fig. 3B, the lesion regions in our study mainly scatter in M5 (*n* = 24), IC (*n* = 24), L (*n* = 18) and M2 (*n* = 16), and it has been demonstrated that IC and M5 behave lower agreement to the ground truth and higher rate of missed diagnosis [52]. Thirdly, to align with the emergency setting, the participant raters in this study are general radiologists rather than neuroradiologists. Compared to the neuroradiologist, the sensitivity and specificity of general radiologist were lower in ASPECTS scoring. This is in line with our findings that the general radiologists performed low sensitivity. At last, this is a multicenter study that have more complicated image quality than a single-center study, which may also weaken the clinical performance of the raters in NCCT-ASPECTS scoring. In a word, compared to previous studies, the external validation data of our study are collected from scanners of multiple vendors, having lower NIHSS and higher ASPECTS. Thus, the diagnostic performance in the external validation set is reasonably acceptable and consistent with reported ones.

Several limitations merit discussion. Firstly, this study mainly focuses on the stroke lesion, neglecting the presence of other neuroimaging signs. Particularly, the existence of leukoencephalopathy and old infarctions may disturb the calculation of ASPECTS. Secondly, the ground truth is MRI infarct images obtained within 24 h after patients receiving complete reperfusion, causing a time delay between the NCCT infarct and the follow-up acquisitions. The time delay may introduce DWI infarct bias as infarct grows over time. Thirdly, patients’ outcome is not taken into consideration yet, and a perspective study ought to be conducted to evaluate the patient’ outcome improvement aroused by the proposed model.

In conclusion, the proposed deep learning model can automatically detect EIC and interpret the ASPECTS and demonstrate improved and reliable performance in the clinical scenario. DL ASPECTS model could be a good assistant to the general radiologist, especially in the hospitals with limited expertise and resource, and further guide the AIS therapeutic decision-making.

## Supplementary Information

**Additional file 1**. This material supplements the details of the image acquisition parameters, model structure, loss function, and self-developed ASPECTS atlas.

## Data Availability

The data that support the findings of this study are available upon request from the corresponding author. The data are not publicly available due to privacy and ethical restrictions.

## Abbreviations

AIS: Acute ischemic stroke
ASPECTS: Alberta Stroke Program Early Computed Tomography Score
AUC: Area under curve
CI: Confidence interval
DCNN: Deep convolutional neural network
DICOM: Digital imaging and communications in medicine
DL: Deep learning
DSC: Dice similarity coefficient
EIC: Early ischemic changes
ICC: Intraclass correlation coefficient
MRI: Magnetic resonance imaging
MRMC: Multi-reader and multicenter
NCCT: Non-contrast computed tomography
RCTs: Randomized controlled trials
RIS: Radiology information system
