# Supplementary material for: Improving the diagnosis of acute ischemic stroke on non-contrast CT using deep learning: a multicenter study
Source: Insights Imaging. 2022 Dec 6;13:184. doi: 10.1186/s13244-022-01331-3 (PMC9723089; doi:10.1186/s13244-022-01331-3)
Supplement: Supplementary file 1 — Additional file 1. This material supplements the details of the image acquisition parameters, model structure, loss function, and self-developed ASPECTS atlas. [file 13244_2022_1331_MOESM1_ESM.pdf]

ELECTRONIC SUPPLEMENTARY MATERIAL

Improving the Diagnosis of Acute Ischemic Stroke on Non-Contrast CT using Deep Learning: A Multicenter Study

Supplementary Material A

Image acquisition parameters

All images were acquired using brain CT protocols on scanners from various vendors, with the X-ray tube voltage of ~120 kV and current of ~400 mA. The matrix size was 512 × 512 and most of the scans had a slice thickness of 5 mm.

DWI was performed on 1.5T or 3T MRI system. All the images were obtained with diffusion gradient b value of 0 and 800 s/mm^2 and/or 1000 s/mm^2.

Supplementary Material B

Supplementary Figure S1: overview of the Global Path: a ResNeXt-based Model

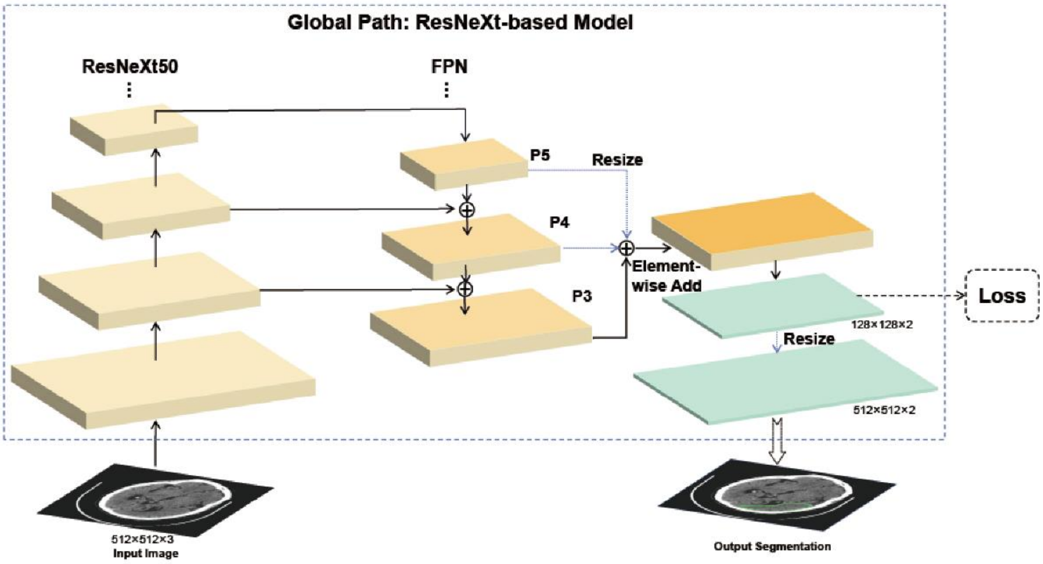

Supplementary Figure S1: We used ResNeXt-50 and FPN architecture as the backbone of the ResNeXt-based Model. The model adopted a top-down network architecture with lateral connections used to build feature maps of different sizes with high-level semantic information. Typically, the high-level semantic features contain a larger receptive field and can capture global information. In order to improve the ability to identify acute ischemic lesions in NCCT images, P4 and P5 were firstly re-sized to the same size with P3 by bilinear interpolation, and then we aggregated high-level semantic feature layers P3, P4 and P5. Specifically, we also resized the input samples to the same size of P3 and calculated the loss in the stage. Note that in the development of the model, our priority was to locate the lesion in a time-effective manner rather than pursuing accurate segmentation.

Supplementary Figure S2: overview of the Local Path: a Dense Unet-based Model

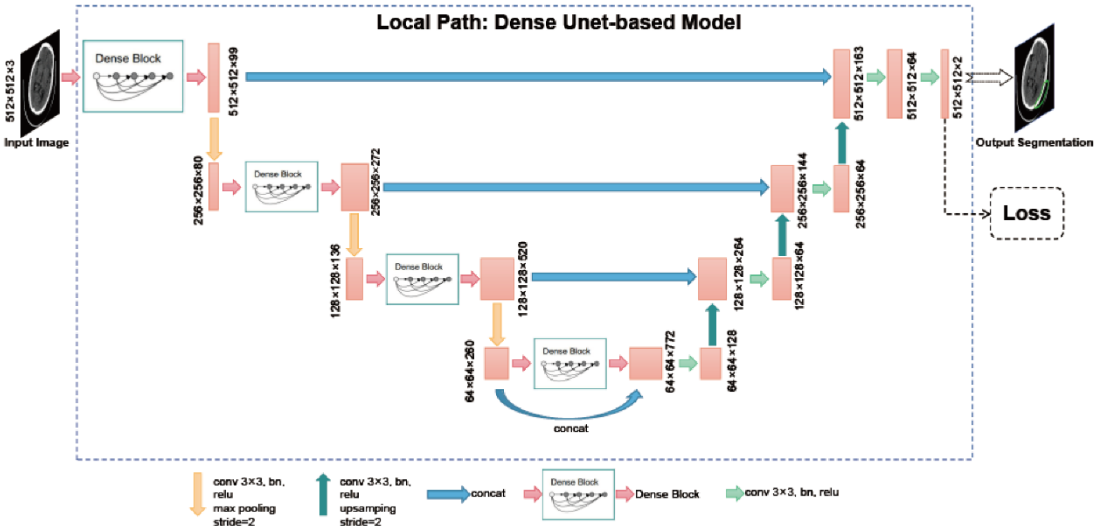

Supplementary Figure S2: The Dense Unet-based model was implemented with a dense connection (“dense block”) and a Unet network architecture as its feature extraction module. The model network structure is shown in Fig. 2, which forms the Unet architecture by continuously and successively stacking a convolution layer, a "relu" activation layer, a max pooling layer and a dense block. The left part of the model underwent *m* down-sampling process, while the right part takes *m* up-sampling process (where *m*=1,2,3...). Moreover, skip connections were used to connect the feature maps between the left and right parts by using the “Concat” operator. Notably, Dense Block retains the shallow features in the down-sampling processes – similar to the spatial information – and reduces the difficulty of convergence. In the up-sampling process, skip connections were performed in multiple scales, enabling the model to evaluate the initial estimate and features of the entire image, and greatly improve the model expression ability.

Loss Function

In order to make the model focus simultaneously on the details as well as the overall segmentation contour of the lesion, we constructed a loss function integrating the cross entropy and DICE loss functions. The formulas are as follows:

$$L = \alpha L_{bce} + (1-\alpha) L_{dice} \quad (1)$$

$$L_{bce} = \sum_{n=1}^{N} -[p_n \log r_n + (1-p_n)\log(1-r_n)] \quad (2)$$

$$L_{dice} = 1 - \frac{\sum_{n=1}^{N} p_n r_n + \varepsilon}{\sum_{n=1}^{N} p_n + r_n + \varepsilon} - \frac{\sum_{n=1}^{N} (1-p_n)(1-r_n) + \varepsilon}{\sum_{n=1}^{N} 2 - p_n - r_n + \varepsilon} \quad (3)$$

where P represents the predicted segmentation, p_n represents the prediction value corresponding to the n-th pixel in P, P* is the gold standard marked by the experts, and r_n represents the label value (∈{0,1}) corresponding to the n-th pixel in the gold standard P*. In addition, α (∈[0,1]) is a modulation parameter, and ε is a small smooth coefficient.

Supplementary Figure S3: the axial slices of self-developed ASPECTS atlas

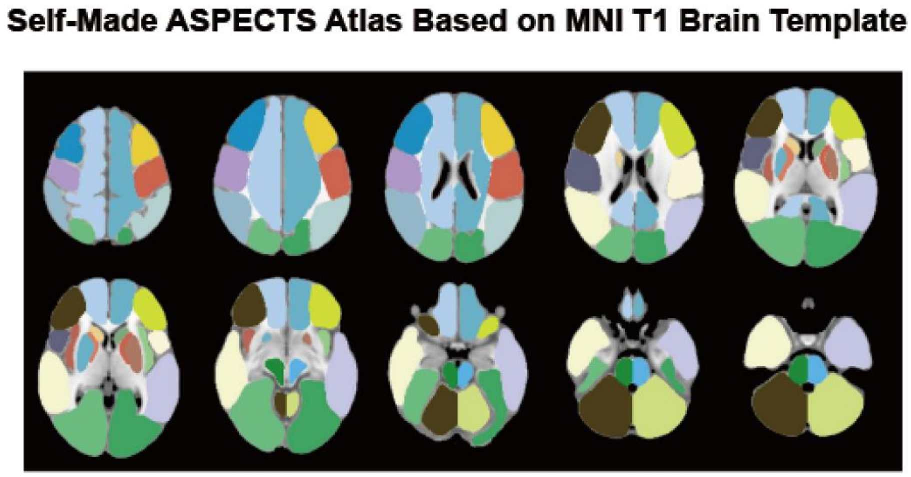

Supplementary Figure S3: The self-developed ASPECTS atlas was built based on the MNI152 brain T2 template with blood supply region labeled. The blood supply regions were segmented manually using 3D Slicer by three board-certified radiologists.
